# Food and Nutrient Intake and Nutrient Sources in 1-Year-Old Infants in Finland: A Cross-Sectional Analysis

**DOI:** 10.3390/nu9121309

**Published:** 2017-12-01

**Authors:** Helena H. Hauta-alus, Liisa Korkalo, Elisa M. Holmlund-Suila, Jenni Rosendahl, Saara M. Valkama, Maria Enlund-Cerullo, Otto M. Helve, Timo K. Hytinantti, Outi M. Mäkitie, Sture Andersson, Heli T. Viljakainen

**Affiliations:** 1 Children’s Hospital, University of Helsinki and Helsinki University Hospital, Biomedicum 2C, P.O. Box 705, 00020 HUS Helsinki, Finland; elisa.holmlund@helsinki.fi (E.M.H.-S.); jenni.rosendahl@hus.fi (J.R.); saara.valkama@hus.fi (S.M.V.); maria.enlund-cerullo@hus.fi (M.E.-C.); otto.helve@helsinki.fi (O.M.H.); timo.hytinantti@hus.fi (T.K.H.); outi.makitie@helsinki.fi (O.M.M.); sture.andersson@hus.fi (S.A.); 2 Division of Nutrition, Department of Food and Environmental Sciences, University of Helsinki, P.O. Box 66, 00014 Helsinki, Finland; liisa.korkalo@helsinki.fi (L.K.); heli.viljakainen@helsinki.fi (H.T.V.); 3 Folkhälsan Research Center, Haartmaninkatu 8, P.O. Box 63, 00014 Helsinki, Finland; 4 Center for Molecular Medicine, Karolinska Institute and Clinical Genetics, Karolinska University Hospital, Solna, 171 76 Stockholm, Sweden

**Keywords:** infant nutrition, breastfeeding, food consumption, nutrient intake

## Abstract

The infant diet has short- and long-term health consequences. Updated data regarding the dietary intake of Finnish infants are lacking. The objectives of this study were to describe infant food and nutrient intake and to identify food sources of the nutrients. Altogether, 739 healthy infants were studied. Dietary intake and breastfeeding frequency were assessed with a three-day food record at 1 year of age. Dietary intake was calculated separately for non-breastfed and breastfed infants. One-third (36%) of the infants were partially breastfed and 95% consumed mass-produced baby foods. The infants’ diet consisted mainly of infant formula, dairy milk, porridges, fruit and berry foods, and meat dishes. The mean vegetable, fruit and berry consumption was 199 g/day. Most nutrient intakes were adequate except for fat, linoleic acid, vitamin D and iron from food. Mean sucrose intake, as a percentage of total energy intake (E%), was 5–6 E%. High protein intake (>20 E%) was observed in 19% of non-breastfed infants. Overall, the infants’ diet was favorable since vegetable and fruit consumption was reasonably high and nutrient intake was mostly adequate. However, the fat intake was lower, and protein intake higher than recommended. Increasing the consumption of vegetable oils and reducing the intake of red meat and dairy milk may further improve the diet of 1-year-olds.

## 1. Introduction

Poor diet is a major contributing factor for many chronic diseases. The origin of many chronic diseases may be traced back to childhood diet [1,2,3]. Suboptimal diet in early life can lead to several unfavorable metabolic conditions through epigenetic programming, and have consequences on health outcomes later in life [4,5]. In fact, early life dietary habits associate with cardiometabolic risk factors in childhood [6,7], and also later in adulthood [3]. Moreover, dietary habits adopted in childhood often persist into later life [8,9]. Evidently healthy dietary habits and adequate nutrient intake are vital for the optimal growth and development of children.

Based on the latest national FINDIET 2012 survey among Finnish adults, a previously observed positive development in dietary habits has partially plateaued [10]. This survey demonstrated that protein and saturated fat intake had increased [10]. However, consumption of vegetables and fibre intake had also increased [10]. The popularity of low carbohydrate diets may be the underlying reason for these partly adverse changes in the diet. Indeed, people following a low carbohydrate diet had unfavorable fat intake and blood lipid profiles [11]. Regarding children, mounting evidence indicates that high protein intake in infancy stimulates growth and body mass index (BMI) accrual in childhood [12,13,14,15,16,17,18,19].

In Finnish children, low intake of fruits and vegetables [20], dietary fibre [21], vitamin D, and iron [22], and high intake of saturated fats and sucrose [22] have been a concern. Low intakes of vitamin D and iron have been observed in Swiss [23], British [24] and US children [25]. Due to changes in national health policies in 2010, vitamin D intake has increased and the prevalence of vitamin D deficiency has decreased in adults [26], pregnant women and newborns [27]. The latest infant dietary data in Finland dates back to 2003–2004 [22,28]. New national food recommendations for children were published in 2016 [29]. Updated evaluation of infant food consumption and nutrient intake is needed in order to promote healthy eating patterns in infants and their families. The objectives of this study were to describe food and nutrient intake and to identify the major food sources of nutrients whose intake may be of concern in 1-year-old infants.

## 2. Methods

### 2.1. Subjects

At Kätilöopisto Maternity Hospital in Helsinki, Finland, 987 families were recruited into the Vitamin D intervention in Infants study (VIDI) between January 2013 and June 2014. Infants were randomized to receive daily vitamin D_3_ supplementation of either 10 μg or 30 μg from 2 weeks old until 2 years of age [30]. A detailed description of the recruitment and study protocol have been reported elsewhere [30]. For the present cross-sectional study, we included 1-year-old infants with food records. Infants with incomplete food records (*n* = 8) and food records which were conducted more than two months after the age of 1.0 year were excluded (*n* = 15). This resulted in total 739 infants, of whom 365 (49%) were girls.

Written informed consent was obtained from the parents at recruitment. This study was conducted according to the guidelines laid down in the Declaration of Helsinki. Ethical approval was obtained from the Research Ethics Committee of the Hospital District of Helsinki and Uusimaa (107/13/03/03/2012). The project protocol is registered in ClinicalTrials.gov (NCT01723852). 

### 2.2. Dietary Intake

Infant food consumption data at 1 year of age were collected between January 2014 and June 2015 with a three-day food record administered to parents or daycare personnel, who had received written instructions on how to accurately fill-in the food record. At 1-year follow-up visits, families were instructed to record all of the infant’s foods, drinks and supplements with the amounts and detailed descriptions of the food items (e.g., brand name, ingredients used and preparation method), as well as breastfeeding frequencies during the food record period. The volume of breast milk ingested was not recorded. The portion size was estimated with household measures, weighed on a home-based scale or recorded from the food package labels. Food records were instructed to comprise, consecutively, two weekdays and one weekend day. Completed food records were reviewed by a nutritionist, and any missing information was requested from parents. 

Food records and nutrient intakes were processed by nutritionists using AivoDiet software (version 2.0.2.3, Aivo Oy, Turku, Finland), which utilizes Fineli (version Fine68, National Institute for Health and Welfare, Helsinki, Finland, 2016), the National Food Composition Database maintained by the National Institute for Health and Welfare. Food and nutrient intake was calculated as a daily mean or median intake from the total food record period. Some home-cooked dishes were recorded as both a recipe and complemented with a separate food item. In a few cases, this separate food item was recorded as fresh vegetable because cooked vegetable was not an available choice in the database, thus the consumption of fresh vegetables might be slightly over-reported. 

Five infants had a two-day, and the rest of the infants (*n* = 734) had a three-day food record. Infants were categorized into two groups, non-breastfed and breastfed infants, based on breastfeeding during the food record period. All breastfed infants were partially breastfed. Categorizing the food items into more general food groups was based on the FINDIET survey [10], with some modifications; for example, we added food groups such as mass-produced baby foods, infant formula and different porridges. Food categorizing was based on culinary criteria; for example, avocados were considered vegetables. Fruit juices were included in the ‘fruit and berry foods’ food group. The ‘vegetables, fruits and berries’ food group consisted of fresh and cooked foods, but not fruit juices. Also food group ‘fresh fruits’ did not include fruit juices. Items from the food record which were composite foods or dishes were unable to be further broken down into individual food items for this analysis. Thus, the total intake of a specific food item, for example meat or vegetables, is unknown, and the ‘meat dishes’ food group includes all ingredients used in the dish, such as meat, potatoes and carrots. Food ‘consumers’ represent those infants who ate the food(s) within the food group during the food record period.

The nutrient content of breast milk was not included in the analyses, as we could not reliably estimate the volume of breast milk. In addition, the nutrient content of vitamin D supplements or other food supplements were not included in the analyses. Sucrose intake contained both the added sucrose in foods and the naturally-occurring sucrose in foods like fruits and berries. The nutrient and food intake was compared with the Nordic Nutrition Recommendations [31], the dietary reference values from the European Food Safety Authority (EFSA) [32,33,34] or the Institute of Medicine (IOM) [35], and national food recommendations [29]. 

### 2.3. Demographic Variables

Parental data were obtained from self-administered questionnaires or from medical records. Parental education level was categorized into ‘lower’ and ‘higher’ education (lower = lower or upper secondary or post-secondary non-tertiary education/less than a bachelor degree, higher = first or second stage of tertiary education/at least a bachelor degree), according to the highest received degree of the parents. Parental smoking status was defined with a dichotomous variable merging previous (pre-pregnancy) and current smoking status, which were assessed with questionnaires at study onset and when the child was two years old. Family income level was gathered with a questionnaire completed at child age of two years. The number of siblings was asked from parents at the 1 year follow-up visit.

### 2.4. Anthropometric Variables

Infant weight (kg) and length (cm) were measured at 1 year follow-up by a pediatrician or a research nurse. Length was measured with a tabletop meter in a supine position, and weight with a scale (Seca^®^, Hamburg, Germany). Length was expressed as standard deviation scores (SDS) and weight as a length-adjusted weight percentage using age- and sex-specific Finnish references [36]. Normal length and weight were considered between −2.0 and +2.0 SDS, and −10.0 and +10.0 percentage, respectively.

### 2.5. Data Analyses

The normality of the variables was visually inspected. Intakes of foods and nutrients were reported as means, medians, standard deviations, and percentages of food consumers. Key background characteristics were compared between non-breastfed and breastfed 1-year-old infants with the independent samples *t*-test, Mann–Whitney *U*-test or Chi-Square test when applicable. In food consumption analyses, the proportion of consumers were compared between non-breastfed and breastfed infants with the Chi-Square test. Food intakes were compared between all non-breastfed and breastfed infants and separately among food consumers with the Mann–Whitney *U*-test. Differences were considered significant at *p* < 0.05. All statistical analyses were conducted using the IBM SPSS program for Windows, version 22 (IBM, Chicago, IL, USA). 

## 3. Results

Of the infants, 97% were cared for at home and 3% at daycare. A total of 36% (263/739) of the infants were partially breastfed at 1 year of age. Mean (SD) daily breastfeeding frequency was 4 (3) with a mean range varying between 0.3 and 16 among breastfed infants (*n* = 263). Of the breastfed infants, 35% received breast milk less than three times per day. Those infants who received breast milk but not infant formula comprised 29% (217/739), 34% (252/739) received infant formula but not breast milk, 6% (46/739) received both, and 30% (224/739) did not receive breast milk or infant formula. In addition, 74% of the infants drank as such dairy milk, of which 4% was whole milk, and the rest was low-fat or skimmed milk. Besides vitamin D supplements (the study preparation for the intervention trial), 11% took food supplements during the food record period, the most common of which were probiotics (10%). 

Socio-demographic characteristics are presented in Table 1. Breastfed infants had lower weights and length-adjusted weights and were more likely to have siblings than those who were not being breastfed. Parents of breastfed infants were older, more highly educated, less likely to smoke, and had lower income levels, compared with non-breastfed infants. The association between lower income level and breastfeeding remained after we included only those infants who were in home care (*X*^2^ Test, *p* = 0.019).

### 3.1. Food Consumption

The majority of infants (95%) consumed mass-produced baby foods during the food record period. The daily mean and median food intake and proportion of food consumers are presented in Table 2 for non-breastfed and breastfed infants separately. Dairy milk, infant formula, porridges, fruit and berry foods and meat dishes were among the most consumed foods (Table 2). In the whole group, the mean (SD) daily intake of combined vegetables, fruits and berries was 199 (104) g. The mean intake of fresh vegetables and fresh fruits was 23 (34) g and 60 (60) g, respectively. The majority of infants ate at least some fresh vegetables (73%) and fruits (84%). Berries were consumed by 38% of infants, and the mean daily intake of berries as such was 9 (18) g in the whole group. Water, infant formula and dairy milk were the main drinks in the population, since the volume of breast milk was unknown. Non-breastfed and breastfed infants consumed, on average, 494 g/day and 150 g/day of dairy products, respectively. Daily mean (SD) frequency of red meat consumption was 1.0 (0.6) (*n* = 739).

Compared with breastfed infants, non-breastfed infants ate less fresh vegetables, berries, egg dishes and plant-based milk products, and more porridges, meat dishes, infant formula and dairy milk (Table 2). Furthermore, in a subgroup of food consumers (those who ate the food), we observed that non-breastfed infants ate less vegetable dishes and more porridges, meat dishes, infant formula and dairy milk compared with breastfed infants (Supplemental Table S1). Seasonal variability of food consumption was small, however, due to seasonal availability, the intake of berries was higher in summer than in other seasons (ANOVA, *p* < 0.001).

### 3.2. Nutrient Intake

The mean (SD) daily nutrient intakes in non-breastfed and breastfed infants are shown in Table 3. The intake of most nutrients from food was adequate in the 1-year-old infants. However, intakes for fat, linoleic acid, vitamin D and iron from food were inadequate in both groups. The mean protein intake was higher and total fat intake lower than recommended [31]. The mean intake of saturated fat was higher than recommended in non-breastfed infants. Among non-breastfed infants, protein intake from total energy intake (E%) was high (>20 E%) in 19%, and fat intake was low (<30 E%) in 52% [35]. The total intake of sucrose did not exceed the recommended daily allowance for added sugar intake. Dietary fibre intake was in line with current recommendation. The mean daily vitamin D intake from food was 7.5 µg (range: 0.6–28.3 µg) in non-breastfed and 3.8 µg (range: 0–30.7 µg) in breastfed infants. Among breastfed infants, frequency of breastfeeding correlated inversely with intakes of all nutrients that were studied here (*r* > 0.23, *p* < 0.001).

### 3.3. Food Sources of Macronutrients, Vitamin D and Iron

Food sources of macronutrients, vitamin D and iron are listed in Supplemental Table S2. In general, dairy products (especially infant formula), meat dishes and porridges were the key sources of several nutrients. Plant-based milk products as a source of nutrients contributed to less than 3% for every nutrient examined. The main food sources of protein and fat were dairy products and meat dishes. The principal food sources of carbohydrates were porridges and dairy products for non-breastfed infants and porridges and fruit and berry foods for breastfed infants. The main sources of vitamin D were dairy products. In addition, milk-based and mass-produced baby food porridges were among the top sources of vitamin D, accompanied with fish dishes in breastfed infants. The key source of iron were porridges, as well as dairy products for non-breastfed and meat dishes for breastfed infants.

## 4. Discussion

The aim of the current study was to describe food and nutrient intake in 1-year-old infants to better understand the current trends in infant feeding and identify possible concerns. The majority of infants studied here were cared for at home, and 36% were partially breastfed at the age of 1 year. The infants’ diet consisted mainly of dairy milk, porridges, fruit and berry foods and red meat dishes, as well as infant formula in non-breastfed infants. Mass-produced baby foods were commonly used, hence, the infant diet had not yet transitioned to the regular family diet. Vegetable, fruit and berry consumption, including the intake of fresh vegetables and fruits, was relatively high in this study population. Food consumption differed according to breastfeeding status at 1 year of age: the diet of breastfed infants was more vegetable- than meat-based compared with non-breastfed peers. Sucrose intake was in line with recommendation and the dietary intake of most nutrients was adequate, except for fat, linoleic acid, vitamin D and iron from food. Interestingly, the intake of protein exceeded the recommendations.

In Finland, breastfeeding is recommended until 1 year of age, or longer if the family prefers [29]. In our study population, 36% of the 1-year-old infants were partially breastfed, which is in accordance with national data from 2011 [37]. In general, the families in our study had higher education level and higher income level compared to the average national level [38,39]. Here, the breastfed infants’ parents were older and more educated, but their income level was lower than non-breastfed infants’ parents. Sociodemographic factors are known to influence infant feeding patterns [40]. In Finland, the mother typically uses both paid maternity and parental leave until the infant is 9 months old, and after this, child home care allowance can be granted for a caregiver until the child is three years of age. The association between lower family income level and breastfeeding was unexpected. 

The breastfed infants’ food intake differed from non-breastfed infants, and it can be argued that the breastfed infants’ dietary pattern was more health conscious or vegetarian-oriented, while the non-breastfed infants’ diet was more traditional Finnish. Resembling differences in food consumption according to breastfeeding status have been observed nationally [28] and in the greater European context [41]. Our findings suggest that continuing breastfeeding to 1 year of age relates to distinctive differences in food intake, which, if maintained, might have long-term health consequences. 

Low consumption of vegetables, fruits, and berries among Finnish children has been a common observation [22], and several studies have shown that the consumption of these foods protect against chronic diseases [3,42]. Fortunately, in this study, the intake of vegetables and fruits in Finnish 1-year-old infants was noticeably higher than reported roughly a decade ago, although the food groups are not fully comparable between studies [22]. Yet, there are no guidelines regarding the amount of vegetables, fruits and berries infants should eat, but, generally, a daily amount of at least 250 g is considered a desirable target for children [29]. In 2003, 1-year-old Finnish infants ate 139–156 g/day of vegetables, fruits and berries [22]. In our study, infants ate, on average, 199 g/day of vegetables, fruits and berries, of which almost half consisted of mass-produced fruit baby foods. However, since this amount does not include composite foods, the actual total amount is even higher. A somewhat similar intake of vegetables and fruits in this age group was found in Ireland (mean intake excluding composite dishes, 162 g/day) [43]. A surprising finding was the reasonably high mean intake of fresh vegetables in 1-year-old infants: 21–25 g/day compared to the previously reported 5–6 g/day [28]. Furthermore, the mean intake of fresh fruits (59–60 g/day) in our study was twice as high as national data from 2004, when the mean intake was 24–25 g/day [28]. 

The frequent consumption of meat dishes, especially red meat dishes, in our study infants can be regarded as a concern due to possibly high content of nitrites/nitrates [44] and protein in red meat products, and acknowledging the observed associations between high consumption of red meat and increased risk of several chronic diseases [45,46]. Albeit, we could not determine the absolute amount of meat consumed. Meat dishes were one of the main sources of protein. Favorably, however, the intake of meat dishes in our cohort was lower than previously reported among 1-year-olds [28]. The current recommendation is to eat white poultry meat 2–3 times per week and red meat less frequently [29]. In our study, almost all infants (87–95%) ate red meat dishes, with less eating poultry dishes (66–69%), during the food record period. On average infants consumed red meat more often than recommended (once a day vs. twice a week) [29]. One approach to decrease red meat intake in infants could be to increase the variety of mass-produced vegetable and fish baby foods compared with red meat baby foods.

Mean protein intake exceeded the recommendation (16.0–16.5 E% vs. 10–15 E%), and was higher than formerly reported in Finnish infants [22]. Similarly, high protein intake has been noted in other European countries as well [47]. We observed that 19% of non-breastfed infants had a mean protein intake above the acceptable intake of >20 E%, set by the IOM [35]. In their review, Hörnell et al. (2013) suggested that the upper limit for mean protein intake for 1-year-old infants should be 15 E%, due to the increased risk for obesity in later life [13]. Protein, especially animal protein, intake in infancy is associated with unfavorable health outcomes in later childhood [15]. The infants’ main protein sources were dairy milk products, meat dishes and porridges. Dairy products have also been the main source of protein in other studies in infants [47]. Non-breastfed infants consumed almost 500 g of fluid dairy milk products daily, while the recommendation is only 400 g [29]. Thus, by reducing dairy milk intake the protein intake would decrease as well.

In line with other studies in small infants, sucrose intake was low in this population, although high variance in the reporting of different sugars complicates comparisons [48]. Our finding of inadequate fat intake was consistent with previous national data [22], as well as a recent Dutch study [49]. The quality of the fat intake, however, was suboptimal; saturated fat intake was higher than recommended in non-breastfed infants, while the intake of essential fatty acid linoleic acid, fell below the recommendation. Since our infants ate porridges regularly, it would be practical to add vegetable oils to porridges as a way to promote good quality fat intake in small infants. 

Vitamin D insufficiency has been the focus of nutritional concerns in Finland for years [26,50]. In order to increase the vitamin D intake, in 2010, vitamin D fortification in Finland was doubled in fluid dairy products and dietary fats, which now contain 1 μg/100 mL and 20 μg/100 g of vitamin D_3_, respectively. Infant formula contains 1.3 µg/100 mL of vitamin D_3_. In addition to food fortification, daily vitamin D supplementation of 10 µg/day is recommended for infants up to the age of 2, and 7.5 µg for children until the age of 17. However, our study infants were given free-off-charge daily vitamin D supplementation dosages of either 10 µg or 30 µg in a double-blinded manner. Compared with findings from 1998–1999 in 1-year-old infants, the mean vitamin D intake from food has improved considerably from 4 µg/day [50] to the current 7.5 µg/day (in non-breastfed infants). Non-breastfed study infants received more vitamin D from food than previously reported from food and supplements combined (6.8 µg/day) [22], however, breastfed infants received only 3.8 µg/day. In line with a previous study, the main food sources of vitamin D were infant formula and dairy milk [28]. Dietary fats only marginally contributed to vitamin D intake because their consumption was low. To conclude, infant vitamin D intake from food has increased, but vitamin D supplementation is still needed to reach the recommended daily amount of 10 µg. 

Parallel to previous studies, from Finland [22] and the UK [24], iron intake did not reach the recommendation in our study. Optimal iron intake is critical for infant development. Since iron deficiency has been less common than inadequate intake of iron among small children in Europe, it has been speculated that the dietary recommendation for iron intake might be overestimated [51]. Our own results support this, as only 2% of infants in the VIDI study had ferritin concentrations below the lower reference range (6 µg/L) [52].

We have collected a large cohort of healthy 1-year-old infants with extensive background details, as well as food record collectively representing year-round food consumption. We herein updated the data on food and nutrient intakes in 1-year-old Finnish infants. However, as our recruitment took place in a single maternity hospital in the capital city of Finland, and the participated families had higher educational and income levels compared with national level, our results may not be generalised to the entire population. A limitation in our study is that we were unable to calculate the absolute amounts of foods that were a part of the composite foods or dishes due to restraints in the software program. Comparing food intakes between studies proved challenging because the basis of the food groupings and reporting may vary. The volume of breast milk was unknown, thus limiting the results of nutrient intake in breastfed infants. Another limitation is that we did not calculate an estimation of usual nutrient intake, taking into account the intra-individual variation; hence, we used mean values of the three-day food record [53,54]. Nevertheless, we did not estimate the proportions of infants with inadequate or excessive micronutrient intakes, only for macronutrients, where the possible bias is likely to be low [54]. As with most dietary methods, the food record might involve problems with under- or over-reporting. Yet, regarding within-subject variation in dietary intake, three-day-food records have been shown to be accurate to estimate nutrient intake (including protein) in 1-year-old infants [55].

Diet quality may decline after the age of 1 year, when children start to adopt the family diet [22]. In our study, infants had not yet transitioned to the regular family diet, since infants were heavily consuming mass-produced baby foods and porridges, and rarely bread and fat spreads. Hence, some unhealthy dietary habits may appear later in childhood, when the diet is less controlled by parents.

Many aspects in the diet of Finnish infants are good, for example, the relatively high intake of vegetables, fruits and berries, the low intake of sucrose, and the adequate intake of a majority of the nutrients. Nevertheless, an inadequate fat intake, especially unsaturated fat might threaten the infants’ health. The infants presented with a high intake of protein and they consumed red meat more frequently than recommended for this age group in Finland. A challenge is to maintain the healthy dietary habits at 1 year of age into later childhood, when the infant further adapts to the family diet. Regular consumption of vegetable oils and a reduction in red meat and dairy milk consumption would optimize the infant diet. 

## Figures and Tables

**Table 1:** Characteristics of non-breastfed (*n* = 476) and breastfed (*n* = 263) 1-year-old infants.

Infant Characteristics	Non-Breastfed	Breastfed	
	Mean (SD)	Mean (SD)	*p* Value ^a^
Infant age, month	12 (0.5)	12 (0.5)	0.560
Length, cm	75.4 (2.6)	75.2 (2.5)	0.152
Length, SDS	−0.5 (1.0)	−0.6 (1.0)	0.147
Weight, kg	9.9 (1.1)	9.6 (1.1)	<0.001
Length-adjusted weight, %	2.4 (8.2)	−0.1 (8.5)	<0.001
Normal length SDS, %	94	93	0.712
Normal length-adjusted weight, %	78	74	<0.001
Gender, girls, %	49	50	0.747
Siblings, % ^b^	31	41	0.022
Maternal age, year	32 (4)	33 (4)	0.026
Paternal age, year ^c^	34 (5)	35 (5)	0.028
Parental education, higher, % ^b^	79	88	0.003
Family income level ^d^			0.009
<40,000 €/year, %	14	23	
40,000–89,000 €/year, %	64	59	
>90,000 €/year, %	22	18	
Maternal smoking, % ^e^	19	8	<0.001
Paternal smoking, % ^e,f^	27	19	0.008

^a^ Independent samples *t*-test, Mann–Whitney *U*-test and Chi-Square test used as applicable; ^b^ 1 value missing; ^c^ 7 values missing; ^d^ 35 values missing and 38 unable to answer; ^e^ previous and current smoking status; ^f^ 7 values missing.

**Table 2:** Mean and median daily food intake (g) and percentages of consumers in non-breastfed (*n* = 476) and breastfed (*n* = 263) 1-year-old infants.

Food Groups, g	Non-Breastfed			Breastfed			Difference in Mean Values
	Mean (SD)	Median	Consumers, %	Mean (SD)	Median	Consumers, %	Sig. ^a^
**Vegetable dishes**	66 (68)	48	90	80 (70)	67	93	*
Fresh vegetables	21 (34)	8	71	25 (32)	13	78	*
Mass-produced vegetable baby foods	14 (32)	0	20	14 (33)	0	21	
**Potato foods**	15 (25)	0	47	13 (19)	1	51	
**Fruit and berry foods**	174 (94)	165	100	172 (78)	160	100	
Fresh fruits	59 (64)	42	82	60 (54)	48	87	
Berries	8 (16)	0	35	12 (20)	0	44	*
Mass-produced fruit and berry baby foods	96 (80)	83	85	93 (76)	80	90	
Fruit juices	2 (13)	0	4	1 (4)	0	3	
**Cereal foods**	36 (35)	26	92	32 (31)	23	94	
Buns and biscuits	3 (8)	0	25	4 (11)	0	26	
**Porridges**	264 (136)	253	98	219 (116)	217	97	*
Water-based porridges	136 (141)	103	69	128 (126)	92	76	
Mass-produced baby food porridges	66 (114)	0	38	43 (84)	0	33	*
**Dietary fats**	3 (5)	1	55	3 (6)	1	58	
**Fish dishes**	31 (40)	12	61	31 (38)	13	67	
Mass-produced fish baby foods	16 (30)	0	25	13 (26)	0	22	
**Egg dishes**	3 (9)	0	15	4 (10)	0	25	*
**Meat dishes**	151 (93)	134	99	121 (84)	110	94	*
Red meat dishes	108 (79)	94	95	82 (70)	69	87	*
Poultry dishes	43 (53)	23	69	39 (49)	17	66	
Mass-produced meat baby foods	90 (89)	67	70	72 (80)	63	63	*
**Dairy and vegetable milk products**	506 (206)	515	100	170 (156)	134	94	*
Infant formula	179 (220)	50	53	31 (93)	0	17	*
Dairy skimmed milk	136 (208)	0	50	29 (75)	0	31	*
Dairy low-fat milk (1.5%)	110 (189)	0	44	35 (74)	0	37	*
Dairy yoghurt	29 (44)	0	50	29 (49)	0	47	
Plant-based milk products	12 (77)	0	4	12 (60)	0	11	*
**Sweets and chocolate**	2 (8)	0	17	1 (2)	0	17	
**Snack foods**	1 (2)	<1	44	1 (3)	1	49	
Unsalted corn or rice snack	1 (2)	0	43	1 (3)	0	47	
**Drinks**	64 (86)	33	65	69 (82)	47	72	
Water	63 (85)	32	64	69 (83)	45	71	

^a^ Sig., a significant difference in mean values tested with Mann–Whitney *U*-test, * indicates a significant association of *p* < 0.05. Dishes include all ingredients used in the recipe.

**Table 3:** Mean daily nutrient intake from food (unless stated otherwise) in non-breastfed (*n* = 476) and breastfed (*n* = 263) 1-year-old infants.

Nutrients	Non-Breastfed	Breastfed	Recommendation ^a^
	Mean (SD)	Mean (SD)	
Energy, MJ	3.7 (0.7)	2.7 (0.8)	
Energy, kcal	881 (168)	654 (201)	
Energy, kJ/kg	375 (79)	287 (92)	333–337
Protein, g	36.3 (10.5)	26.5 (10.4)	11–12 ^b^
Protein, g/kg	3.7 (1.1)	2.8 (1.1)	1.14 ^b^
Protein, E%	16.5 (3.8)	16.0 (3.2)	10–15
Low protein intake (<5 E%), %	0		5–20 ^c^
High protein intake (>20 E%), %	19		
Carbohydrate, g	111.5 (21.8)	82.9 (26.4)	
Carbohydrate, E%	50.8 (5.3)	51.1 (7.2)	45–60
Low carbohydrate intake (<45 E%), %	14		45–65 ^c^
High carbohydrate intake (>65 E%), %	1		
Total sucrose, g	10.7 (5.9)	9.5 (4.3)	
Total sucrose, E%	4.9 (2.5)	6.0 (2.4)	<10 ^d^
Dietary fibre, g	11.4 (4.0)	10.5 (3.6)	10 ^b^
Dietary fibre, g/MJ	3.1 (0.9)	3.9 (1.0)	2 ^b^
Fat, g	28.6 (9.7)	20.8 (8.6)	
Fat, E%	28.9 (6.7)	28.3 (7.0)	30–40
Low fat intake (<30 E%), %	52		30–40 ^c^
High fat intake (>40 E%), %	3		
SFA, E%	10.6 (3.5)	9.2 (3.6)	<10
MUFA, E%	10.7 (2.9)	10.6 (3.4)	10–20
PUFA, E%	5.2 (1.4)	5.4 (1.7)	5–10
*n*-6, E%	4.5 (1.5)	4.1 (1.3)	3
*n*-3, E%	2.1 (0.5)	2.1 (0.7)	0.5
Linoleic acid, g	3.7 (1.8)	2.6 (1.3)	4
α-Linolenic acid, g	0.9 (0.5)	0.7 (0.4)	0.5
Vitamin A, µg RE	542 (260)	426 (221)	300
Vitamin D, µg	7.5 (3.2)	3.8 (3.0)	10
Vitamin E, mg α TE	5.7 (2.2)	4.1 (1.7)	4
Thiamine, mg	0.8 (0.2)	0.5 (0.2)	0.5
Riboflavin, mg	1.3 (0.4)	0.7 (0.4)	0.6
Niacin, mg NE	14.8 (3.7)	10.8 (4.1)	7
Pyridoxine, mg	1.1 (0.3)	0.9 (0.3)	0.5
Folate, µg	122 (32)	97 (37)	60
Vitamin B_12_, µg	2.8 (1.1)	1.8 (1.1)	0.6
Vitamin C, mg	92 (47)	76 (46)	25
Calcium, mg	729 (280)	406 (241)	600
Potassium, g	1.9 (0.5)	1.5 (0.5)	1.4
Phosphorus, mg	803 (253)	553 (226)	470
Magnesium, mg	176 (47)	137 (47)	85
Iron, mg	6.9 (2.0)	5.2 (1.9)	8
Zinc, mg	6.4 (1.5)	4.4 (1.7)	5

MJ, megajoule; kJ, kilojoule, E%, proportion of macronutrient intake from total energy intake; SFA, saturated fatty acid; MUFA, monounsaturated fatty acid; PUFA, polyunsaturated fatty acid; *n*-6, *n*-6 polyunsaturated fatty acid; *n*-3, *n*-3 polyunsaturated fatty acid; RE, retinol equivalent; α TE, alpatocopherol equivalent; NE, niacin equivalent. ^a^ Recommended intake of nutrients of 1-year-olds by Nordic nutrition recommendations [31]; ^b^ Population reference intake of 1-year-olds for protein [32], and adequate intake for dietary fibre, linoleic acid and α-linolenic acid by European Food Safety Authority (EFSA) [33,34]; ^c^ Acceptable macronutrient distribution range or adequate intakes for 1–3 year-olds by Institute of Medicine (IOM) [35]; ^d^ Recommendation is for added sugars, and total sucrose intake contain also naturally occurring sucrose.

## Supplementary Materials

The following are available online at www.mdpi.com/2072-6643/9/12/1309/s1, Table S1: Mean daily food intake (g) of food consumers in non-breastfed and breastfed 1-year-old infants, Table S2: Food sources of macronutrients, vitamin D and iron in non-breastfed (*n* = 476) and breastfed (*n* = 263) 1-year-old infants.
