# Supplementary material for: Food and Nutrient Intake and Nutrient Sources in 1-Year-Old Infants in Finland: A Cross-Sectional Analysis
Source: Nutrients. 2017 Dec 1;9(12):1309. doi: 10.3390/nu9121309 (PMC5748759; doi:10.3390/nu9121309)
Supplement: Supplementary file 1 [file nutrients-09-01309-s001.zip › Supplemental Table S1.docx]

| Supplemental Table S1. Mean daily food consumption (g) among consumers of each food group in Finnish non-breastfed and breastfed 1-year-old infants. | | | | | |
| --- | --- | --- | --- | --- | --- |
|  | Non-breastfed | | Breastfed | |  |
| **Food groups, g** | N | Mean (SD) | N | Mean (SD) | P value ^a^ |
| Vegetable dishes | 428 | 73 (68) | 244 | 87 (69) | 0.003 |
| Fresh vegetables | 337 | 30 (37) | 204 | 32 (34) | 0.074 |
| Mass-produced vegetable baby foods | 97 | 68 (36) | 56 | 67 (39) | 0.475 |
| Potato foods | 223 | 32 (27) | 133 | 26 (20) | 0.087 |
| Fruit and berry foods | 474 | 175 (93) | 263 | 172 (78) | 0.851 |
| Fresh fruits | 391 | 72 (64) | 229 | 69 (52) | 0.733 |
| Berries | 166 | 23 (20) | 115 | 27 (23) | 0.166 |
| Mass-produced fruit and berry baby foods | 405 | 113 (75) | 236 | 103 (73) | 0.101 |
| Fruit juices | 17 | 46 (51) | 9 | 20 (12) | 0.142 |
| Cereal foods | 438 | 39 (35) | 248 | 34 (31) | 0.070 |
| Buns and biscuits | 120 | 8 (9) | 68 | 8 (8) | 0.664 |
| Porridges | 468 | 268 (133) | 256 | 225 (112) | <0.001 |
| Water-based porridges | 329 | 197 (130) | 200 | 168 (119) | 0.016 |
| Mass-produced baby food porridges | 179 | 175 (125) | 80 | 141 (97) | 0.063 |
| Dietary fats | 264 | 5 (6) | 152 | 5 (6) | 0.350 |
| Fish dishes | 289 | 5 (40) | 176 | 46 (38) | 0.173 |
| Mass-produced fish baby foods | 121 | 61 (26) | 58 | 58 (21) | 0.235 |
| Egg dishes | 71 | 17 (17) | 67 | 17 (13) | 0.455 |
| Meat dishes | 470 | 153 (92) | 248 | 129 (81) | 0.001 |
| Red meat dishes | 453 | 114 (78) | 229 | 95 (67) | 0.003 |
| Poultry dishes | 328 | 62 (54) | 174 | 59 (49) | 0.556 |
| Mass-produced meat baby foods | 333 | 129 (79) | 166 | 115 (73) | 0.095 |
| Dairy and vegetable milk products, g | 474 | 508 (204) | 248 | 181 (155) | <0.001 |
| Infant formula | 252 | 339 (194) | 46 | 175 (159) | <0.001 |
| Dairy skimmed milk | 236 | 274 (223) | 82 | 92 (110) | <0.001 |
| Dairy low-fat milk (1.5%) | 208 | 252 (215) | 98 | 94 (96) | <0.001 |
| Dairy yoghurt | 237 | 59 (46) | 123 | 61 (56) | 0.767 |
| Plant-based milk products | 21 | 260 (269) | 29 | 110 (150) | 0.238 |
| Sweets and chocolate | 79 | 10 (18) | 44 | 3 (3) | 0.052 |
| Snack foods | 247 | 3 (4) | 151 | 3 (3) | 0.310 |
| Unsalted corn or rice snack | 207 | 2 (2) | 129 | 3 (3) | 0.178 |
| Drinks | 310 | 99 (90) | 189 | 96 (83) | 0.901 |
| Water | 305 | 98 (89) | 187 | 96 (83) | 0.756 |
| ^a^ Mann-Whitney U-test | | | | | |
