# Supplementary material for: Food and Nutrient Intake and Nutrient Sources in 1-Year-Old Infants in Finland: A Cross-Sectional Analysis
Source: Nutrients. 2017 Dec 1;9(12):1309. doi: 10.3390/nu9121309 (PMC5748759; doi:10.3390/nu9121309)
Supplement: Supplementary file 1 [file nutrients-09-01309-s001.zip › Supplemental Table S2.docx]

| Supplemental Table S2. Food sources of macronutrients, vitamin D and iron in non-breastfed (n=476) and breastfed (n=263) 1-year-old infants. | | | | |
| --- | --- | --- | --- | --- |
|  | Non-breastfed | | Breastfed | |
|  | Mean daily intake | Proportion of daily intake, % | Mean daily intake | Proportion of daily intake, % |
| **Protein, g** |  |  |  |  |
| Dairy and plant-based milk products | 13.7 | 38 | 6.1 | 23 |
| Dairy skimmed milk | 4.2 | 12 | 0.9 | 4 |
| Dairy low-fat milk (1.5%) | 3.4 | 9 | 1.1 | 4 |
| Infant formula | 2.4 | 7 | 0.4 | 2 |
| Cheese | 1.4 | 4 | 1.5 | 6 |
| Meat dishes | 9.0 | 25 | 7.3 | 28 |
| Red meat dishes | 6.2 | 17 | 4.7 | 18 |
| Porridges | 6.3 | 17 | 5.1 | 19 |
| Water-based porridge | 2.1 | 6 | 2.0 | 8 |
| Mass-produced baby food porridges | 1.9 | 5 | 1.3 | 5 |
| Cereal foods | 2.1 | 6 | 1.9 | 7 |
| Fish dishes | 2.0 | 6 | 2.4 | 9 |
| Fruit and berry foods | 1.3 | 4 | 1.3 | 5 |
| Vegetable dishes | 1.2 | 3 | 1.7 | 6 |
| Other | 0.9 | 2 | 0.9 | 3 |
| **Carbohydrates, g** |  |  |  |  |
| Dairy and plant-based milk products | 30.1 | 27 | 9.5 | 12 |
| Infant formula | 13.5 | 12 | 2.3 | 3 |
| Dairy skimmed and low-fat milk | 11.8 | 11 | 3.0 | 4 |
| Meat dishes | 11.5 | 10 | 9.1 | 11 |
| Porridges | 24.8 | 22 | 20.4 | 25 |
| Water-based porridge | 9.4 | 8 | 9.1 | 11 |
| Mass-produced baby food porridges | 8.3 | 7 | 5.9 | 7 |
| Cereal foods | 12.3 | 11 | 10.9 | 13 |
| Bread | 5.5 | 5 | 4.9 | 6 |
| Fruit and berry foods | 22.4 | 20 | 21.7 | 26 |
| Mass-produced fruit and berry baby foods | 12.0 | 11 | 11.6 | 14 |
| Fresh fruits | 8.5 | 8 | 8.3 | 10 |
| Vegetable dishes | 4.4 | 4 | 5.7 | 7 |
| Potato foods | 2.2 | 2 | 2.0 | 2 |
| Fish dishes | 2.3 | 2 | 2.0 | 2 |
| Other | 1.5 | 1 | 1.5 | 2 |
| **Fat, g** |  |  |  |  |
| Dairy and plant-based milk products | 10.7 | 37 | 4.2 | 20 |
| Infant formula | 6.3 | 22 | 1.1 | 5 |
| Low-fat milk (1.5%) | 1.6 | 6 | 0.5 | 3 |
| Meat dishes | 6.4 | 22 | 5.1 | 25 |
| Red meat dishes | 4.9 | 17 | 3.7 | 18 |
| Porridges | 3.8 | 13 | 2.9 | 14 |
| Mass-produced baby food porridge | 1.7 | 6 | 1.1 | 5 |
| Cereal foods | 1.2 | 4 | 1.2 | 6 |
| Fruit and berry foods | 0.8 | 3 | 0.7 | 3 |
| Vegetable dishes | 1.9 | 7 | 2.1 | 10 |
| Fresh vegetables | 1.0 | 3 | 1.0 | 5 |
| Fish dishes | 1.4 | 5 | 1.6 | 8 |
| Dietary fats | 1.8 | 6 | 2.3 | 11 |
| Other | 0.6 | 2 | 0.8 | 4 |
| **Vitamin D, µg** |  |  |  |  |
| Dairy and plant-based milk products | 4.8 | 64 | 1.3 | 35 |
| Infant formula | 2.3 | 31 | 0.4 | 11 |
| Skimmed milk | 1.3 | 17 | 0.3 | 7 |
| Low-fat milk (1.5%) | 0.9 | 12 | 0.3 | 7 |
| Meat dishes | 0.2 | 3 | 0.2 | 5 |
| Porridges | 1.5 | 19 | 1.0 | 25 |
| Mass-produced baby food porridge | 1.0 | 13 | 0.6 | 17 |
| Milk-based porridge | 0.5 | 6 | 0.3 | 8 |
| Fish dishes | 0.6 | 8 | 0.9 | 24 |
| Dietary fats | 0.3 | 3 | 0.3 | 7 |
| Other | 0.1 | 2 | 0.2 | 5 |
| **Iron, mg** |  |  |  |  |
| Dairy and plant-based milk products | 1.5 | 22 | 0.4 | 8 |
| Infant formula | 1.1 | 16 | 0.2 | 6 |
| Meat dishes | 1.1 | 17 | 0.9 | 18 |
| Red meat dishes | 0.9 | 13 | 0.7 | 14 |
| Porridges | 2.3 | 34 | 1.9 | 36 |
| Mass-produced baby food porridge | 0.9 | 13 | 0.6 | 12 |
| Water-based porridge | 0.9 | 13 | 0.9 | 17 |
| Cereal foods | 0.6 | 8 | 0.5 | 10 |
| Fruit and berry foods | 0.6 | 9 | 0.6 | 12 |
| Vegetable dishes | 0.4 | 5 | 0.5 | 10 |
| Fish dishes | 0.2 | 3 | 0.2 | 3 |
| Other | 0.2 | 3 | 0.2 | 4 |
| Percentages may not add up to 100% due to rounding | | | | |
